# Normal reference values of strength in pelvic floor muscle of women: a descriptive and inferential study

**DOI:** 10.1186/s12905-014-0143-4

**Published:** 2014-11-25

**Authors:** Francine Chevalier, Carolina Fernandez-Lao, Antonio Ignacio Cuesta-Vargas

**Affiliations:** Patronato Municipal de Deportes de Torremolinos, Málaga, Spain; Departamento de Fisioterapia, Universidad de Granada, Granada, Spain; Departmento de Fisioterapia, Universidad de Malaga, Málaga, Spain; School of Clinical Sciences of the Faculty of Health at the Queensland University of Technology, Brisbane, Australia

**Keywords:** Pelvic floor muscles, Stress urinary incontinence, Normal reference values, Scoring measures

## Abstract

**Background:**

To describe the clinical, functional and quality of life characteristics in women with Stress Urinary Incontinence (SUI). In addition, to analyse the relationship between the variables reported by the patients and those informed by the clinicians, and the relationship between instrumented variables and the manual pelvic floor strength assessment.

**Methods:**

Two hundred and eighteen women participated in this observational, analytical study. An interview about Urinary Incontinence and the quality of life questionnaires (EuroQoL-5D and SF-12) were developed as outcomes reported by the patients. Manual muscle testing and perineometry as outcomes informed by the clinician were assessed. Descriptive and correlation analysis were carried out.

**Results:**

The average age of the subjects was (39.93 ± 12.27 years), (24.49 ± 3.54 BMI). The strength evaluated by manual testing of the right levator ani muscles was 7.79 ± 2.88, the strength of left levator ani muscles was 7.51 ± 2.91 and the strength assessed with the perineometer was 7.64 ± 2.55. A positive correlation was found between manual muscle testing and perineometry of the pelvic floor muscles (*p* < .001). No correlation was found between outcomes of quality of life reported by the patients and outcomes of functional capacity informed by the physiotherapist.

**Conclusion:**

A stratification of the strength of pelvic floor muscles in a normal distribution of a large sample of women with SUI was done, which provided the clinic with a baseline. There is a relationship between the strength of the pelvic muscles assessed manually and that obtained by a perineometer in women with SUI. There was no relationship between these values of strength and quality of life perceived.

**Electronic supplementary material:**

The online version of this article (doi:10.1186/s12905-014-0143-4) contains supplementary material, which is available to authorized users.

## Background

The International Continence Society (ICS) defines Urinary Incontinence (IU) as “the complaint of involuntary leakage of urine.” Abrams P et al. [1] One of the three described types of UI, Stress Urinary Incontinence (SUI), is considered to be a burden that has a critical impact on the quality of life for women and is a common condition that affects from 20% to 40% of older women: this prevalence increases with the advance of age [2]. SUI is the most common type of UI and involves an objectively demonstrable and involuntary loss of urine that causes a social problem [3]. This loss of urine occurs during efforts, sneezing, coughing, laughing, etc. [1].

The etiology of UI is multifactorial [4]; there are many factors influencing the perception of this complaint as a health problem. These factors are related to age, obesity [5,6], delivery circumstances [5,7], menopause [8] and others conditions.

The strengthening of pelvic floor muscles is one of the first recommendations for the treatment of mild and moderate SUI. Different modalities include pelvic floor muscle training alone or in combination with biofeedback and vaginal cones or balls [9,10]. Training of pelvic floor muscle during SUI has reached success rates of 56% to 75% [11]. According to a Cochrane review, strengthening should be recommended in conservative programs of the first-line of treatments for SUI [12].

Manual palpation is a technique used today by most physiotherapists to assess the correct contraction of the pelvic floor muscles. It was first described by Kegel [9,13] as a method to evaluate the function of the pelvic floor and is considered to be an essential part of the assessment. The assessment of muscular strength and endurance provides information about the severity of muscle weakness and is the basis for the planning of specific exercise programs for each patient. There are several techniques for the evaluation of pelvic floor muscles, which include the aforementioned digital palpation, pressure manometry, electromyography, ultrasound and magnetic resonance imaging [14]. Among them, the use of perineometers or pressure manometers are often some of the most commonly used alternatives, these instruments have proved their reliability [15,14] and should not be used in isolation, but simultaneously with other methods for the correct observation of the contraction [16].

### Objective

The aim of this study was to describe normal reference values of the strength of the pelvic floor muscles in women with SUI. Second, we analysed the relationship between the variables reported by the patients and those informed by the clinicians, and the relationship between instrumented variables and the manual pelvic floor strength assessment.

## Methods

### Participants

Two hundred and eighteen women between 22 and 85 years of age participated in this observational, analytical study. The patients were recruited from the Community Physiotherapy and Sport Center (specifically the Women’s Health Area) after confirming the diagnosis of urodynamic SUI [17] in specialized units. The diagnosis consisted of having: a) no detrusor over-activity, b) a positive cough stress test and c) a pad test with less than 3 g of leakage with a standardised bladder volume of 200 ml [18]. All women had been suffering from SUI for at least six months, and they had all been examined for SUI. Exclusion criteria were: a) having a cognitive disability, b) physical disability, or c) psychiatric limitations that inhibited participation on the study tests. Two physiotherapists from Torremolinos, Málaga (Spain) voluntarily participated in the study.

The Malaga University Ethics Committee, following the Helsinki declaration, gave ethical clearance for the study. All participants in this study signed an informed consent form before their inclusion.

### Outcomes reported by the patient

#### Interview about urinary incontinence (ad hoc)

Questions were asked about gender, height and weight, and Body Mass Index (BMI) was calculated. A specific clinical interview was developed with the aim of analysing the principal components of pelvic floor dysfunction. A physiotherapist from the Consulting Unit of Physiotherapy of the Pelvic Floor conducted every question. The questionnaire consisted of 38 items, where items 1–11 referred to delivery conditions, items 12–23 presented information about faecal and urinary incontinence and items 24–38 were related to medical conditions and lifestyle (Additional file 1).

Adaptation of Modified Oxford Grading System to evaluate the strength of the pelvic floor muscles by using vaginal palpation by Laycock. This scale was divided in fifteen categories, as follow:

0= “0”: No active muscular contraction

1=”1-”

2=“1”: Very slight muscular contraction

3=“1+”:

4=“2-”

5=“2”: Full-motion overcome the force of gravity

6=“2+”

7=“3-”

8=“3”: Full-motion against gravity

9=“3+”

10=“4-”

11=“4”: Full-motion against slight resistance

12=“4+”

13=“5-”

14=“5”: Full-motion against strong resistance

15=“5+”

#### EuroQol 5D (EQ-5D) questionnaire

The EQ-5D questionnaire is a widely used tool consisting of non-specific illness questions to evaluate the quality of life related to health [19]. It is composed of two parts. Part I: (auto-informed) consists of health problems related to mobility, self-care, usual activities, pain/discomfort and anxiety/depression. Each dimension has three levels: no problems, some problems or extreme problems. Answers are given related to the day when patient completes the questionnaire. Part II: a self-rated Visual Analogue Scale (VAS), where the endpoints are labelled ‘Best imaginable health state’ (100) and ‘Worst imaginable health state’ (0). The EQ-5D has a reliability score between 0.86 and 0.90, and was validated in Spanish by Badia et al. [19].

#### SF-12 health survey scoring demonstration

The SF-12 is a generic instrument that asks questions about quality of life. It consists of a subset of 12 items from the SF-36 selected by multiple regression (two elements from each dimension of physical functioning, physical role, emotional role and mental health, and an element from each dimension of bodily pain, general health, vitality and social functioning) from which the physical and mental component of the SF-12 scores were constructed. The questionnaire has demonstrated a reliability score of 0.70 [20], and the Spanish version was validated by Vilagut et al. [21].

### Outcomes informed by the clinician

#### Manual muscle testing

Laycock [18] developed the Modified Oxford Grading System to evaluate the strength of the pelvic floor muscles by using vaginal palpation. It consists of a six-point scale: 0 = no contraction, 1 = flicker, 2 = weak, 3 = moderate, 4 = good (with lift) and 5 = strong. This measurement scale is widely used by physiotherapists since it can be used with vaginal palpation in the clinical evaluation. For its correct use, manual skill of the physiotherapist is considered essential. All assessments were carried out for the same physiotherapist (FC). It is an easy method to use and does not require expensive equipment [14]. Inter-rater reliability for vaginal palpation was high (*κ* =0.33, 95% confidence interval 0.09 to 0.57) [22].

#### Perineometer

We used a vaginal pressure device connected to a pressure manometer that shows the air pressure through an arbitrary scale from 0 to 12 (PFX 2-Pelvic Floor Exerciser Biofeedback [Cardio Design, Australia]) to assess the strength of pelvic floor muscles [23]. The perineometer reliability was studied by Isherwood and Rane [24] by comparing results with vaginal palpation using the Modified Oxford Grading System. They found high reliability with a kappa value of 0.73.

### Statistical analysis

The descriptive analysis was developed by calculating the means and standard deviations for quantitative variables. For the analysis of categorical variables, we calculated the absolute frequencies and quartiles. The normality of the variables was assessed by the Kolmogorov-Smirnov test. The Pearson correlation coefficient was calculated to test the association between the variables. All tests were interpreted as statistically significant when *p* < 0.05. IBM SPSS Statistics (version 22.0) for Windows was used for this statistical analysis.

## Results

The study sample consisted of 218 women aged 28 to 85 years (35.42 ± 9.30). The main characteristics of the patients were presented together with the results concerning the study of quality of life in Table 1.

**Table 1:** **Sociodemographic anthropometric and quality of life characteristics**

**n = 212**	**(m ± SD)**
**Age (years)**	39.93 ± 12.27
**Weight (Kg)**	70.75 ± 25.94
**Height (cm)**	164.48 ± 7.41
**BMI**	24.49 ± 3.54
**EuroQol (VAS)**	69.32 ± 17.23
**EuroQol (Index)**	0.85 ± 0.18
**SF-12 PCS**	47.54 ± 10.14
**SF-12 MCS**	48.90 ± 9.73
**Physical Function**	50.10 ± 10.54
**Physical Role**	38.92 ± 12.15
**Bodily Pain**	49.89 ± 9.62
**General Health**	50.17 ± 6.72
**Vitality**	47.74 ± 10.44
**Social Function**	51.70 ± 8.10
**Emotional Role**	38.06 ± 14. 49
**Mental Health**	40.02 ± 12.14

Values expressed as mean ± Standard Deviation.

SF-12 PCS. Physical Component Summary.

SF-12 MCS. Mental Component Summary.

Regarding the gynaecological screening, the results were as follows. One hundred-thirteen (53.6%) patients had bladder prolapse, while the remaining 98 (46.4%) did not; 92 (43.6% ) women had womb prolapse, and 119 (56.4%) did not; the presence of rectum prolapse was evident in 41.2% (87 patients).

The results concerning the manual muscle test were the following: the strength of the right levator ani muscles (SD 7.79 ± 2.88), the cut-off points were 6, 7 and 10 for percentile 25, 50 and 75, respectively. The distribution of four categories was 60 (27.9%) in the category very weak, 39 (17.8%) in the category weak, 37 (17.8%) in the category strong and 80 (37.5%) in the category very strong. The strength of left levator ani muscles was 7.51 ± 2.91. The cut-off points were 4, 7 and 10 for percentile 25, 50 and 75, respectively. The distribution of 4 categories was 64 (30.8%) in the category very weak, 41 (18%) in the category weak, 46 (20.3%) in the category strong and 64 (30.8%) in the category very strong.

According to the assessment with the perineometer, the patient group showed strength (7.64 ± 2.55). The cut-off points were 6, 8 and 10 to percentile 25, 50 and 75, respectively. The distribution of the sample categories was 55 (26.1%) in the category very weak (quartile 4), 21 (1.4%) in the category weak, 69 (36.2%) in the category strong. Finally, 169 (82.4%) women had automation effort, versus 35 (17.1%) women who did not.

The results concerning the SUI questionnaire about the deliveries, urinary and faecal incontinence and the information catalogued as heterogeneous, are presented in Tables 2, 3, and 4, respectively.

**Table 2:** **Items’ 1–11 IU questionnaire (deliveries information)**

**n = 212**			
**Mean pregnancies (SD)**	1.89 ± 1.21		
**Mean deliveries (SD)**	1.56 ± 0.84		
**Mean weight (gr) (SD)**	**1** ^**st**^ **labour**	**2** ^**nd**^ **labour**	**3** ^**rd**^ **labour**
	3327.43 ± 1.19	3422.72 ± 533.12	3468.04 ± 58.14
**Type of delivery 1 (%)**	**Normal**	**Cesarean**	**Instrumented**
	125 (64.4%)	34 (17.5%)	35 (18%)
**Type of delivery 2 (%)**	**Normal**	**Cesarean**	**Instrumented**
	65 (78.3%)	10 (12.0%)	8 (9.6%)
**Type of delivery 3 (%)**	**Normal**	**Cesarean**	**Instrumented**
	22 (81.5%)	5 (18.5%)	0 (0%)
**Multiple deliveries (%)**	**Yes**		**No**
	5 (2.45%)		201 (97.55%)
**Episiotomy (%)**	**Yes**		**No**
	149 (73%)		54 (26.6%)

Values expressed as mean ± Standard Deviation (95% Confidence Interval) and absolute percentages.

**Table 3:** **Items 12–23 UI questionnaire (Urinary and fecal incontinence)**

**n = 212**			
**Micturitions/day (SD)**	7.40 ± 2.45		
**Micturitions/night (SD)**	0.80 ± 1.17		
**Volume of fluid ingested/day (%)**	**<1 l**	**1 l**	**>1 l**
	20 (9.3%)	44 (20.3%)	152 (70.4%)
**Reduction of liquid ingested (%)**	**Yes**	**No**	
	69 (31.8%)	148 (68.2%)	
**Suffer loss of urine (%)**	**Yes**	**No**	
	89 (41.4%)	126 (58.6%)	
**Loss during strain or stress (%)**	**Yes**	**No**	
	100 (46.3%)	116 (53.7%)	
**Loss during:**			
**a) coughing. laughing or jumping (%)**	**Yes**	**No**	
	147 (68.1%)	69 (31.9%)	
**b) listening the sound of water (%)**	**Yes**	**No**	
	121 (56.3%)	94 (43.7%)	
**c) after the coition (%)**	**Yes**	**No**	
	95 (44.6%)	118 (55.4%)	
**Feeling strong and imperiously desire of urinate (%)**	**Yes**	**No**	
	106 (49.1%)	110 (50.9%)	
**Need to use pads (%)**	**Yes**	**No**	
	88 (40.7%)	128 (59.3%)	
**Number of pads/day (SD)**	2.38 ± 1.147		
**Suffer constipation (%)**	**Yes**	**No**	
	118 (54.4%)	99 (45.6)	
**Suffer fecal incontinence (%)**	**Yes**	**No**	
	66 (30.4%)	150 (69.1%)	
**Urodynamic test (%)**	**Yes**	**No**	
	88 (40.6%)	129 (40.4%)	

Values expressed as mean ± Standard Deviation (95% Confidence Interval) and absolute percentages.

**Table 4:** **Items 23–38 UI questionnaire (heterogeneous)**

**n = 218**								
**Occupation (%)**	**Standing**	**Sitting**	**Carrying weights**	**All**	**Stand + Weight**	**Stand + Sitting**	**Not clear**	**Sitting + Weight**
	31 (14.4%)	48 (22.2%)	1 (0.5%)	31 (14.4%)	53 (24.5%)	40 (18.5%)	2 (0.9%)	10 (4.6%)
**Quality of life affectation (%)**			**Yes**		**No**			
			122 (50.6%)		94 (43.5%)			
**Comments with relatives/friends (%)**			**Yes**		**No**			
			137 (63.4%)		79 (33.6%)			
**Comments with a specialist (%)**			**Yes**		**No**			
			185 (87.3%)		27 (12.7%)			
**Type of specialist (%)**			**Gynecologist**	**Matron**	**Family doctor**			**Others**
			96 (57.2%)	74 (40.7%)	4 (1.8%)			8 (3.7%)
**Urgency of micturition (%)**			**Yes**		**No**			
			113 (52.3%)		103 (47.7%)			
**Heritage (prolapse, UI, hysterectomy) (%)**			**Yes**		**No**			
			147 (68.1%)		69 (31.9%)			
			**Can not contain gas**		**Can not contain the sediments**			
**Anal incontinence (%)**			55 (25.3%)		162 (74.7%)			
**Sufferings during coition:**								
**Pain (%)**			**Yes**		**No**			
			117 (55.7%)		93 (44.3%)			
**Loss of urine (%)**			**Yes**		**No**			
			78 (37.3%)		131 (62.7%)			
**Feeling of abdominal heaviness (%)**			**Yes**		**No**			
			128 (59.5%)		87 (40.5%)			
**Important diseases**	**Cardiovasc**	**Cancer**	**Psycol Dis**	**Musculosk**	**Others**	**Different types**		**No disease**
	14 (6.5%)	15 (6.9%)	0 (0%)	30 (13.9%)	105 (48.6%)	30 (13.9%)		21 (9.7%)
**Surgical proced.**	**Reumatol**	**Oncol**	**Urol/Gine**	**Cardiov**	**Others**	**Different types**		**No surgery**
	5 (2.3%)	9 (4.1%)	10 (4.6%)	1 (0.5%)	134 (61.8%)	43 (19.8%)		15 (6.9%)
**Menopause (%)**			**Yes**		**No**			
			79 (37.3%)		133 (62.7%)			
**Hormonal treatment (%)**			**Yes**		**No**			
			19 (23.2%)		63 (76.8%)			
**Age of menopause (D.E.)**			47.23 ± 8.05					
**Physical exercise (%)**			**Pelvic floor impact**		**No impact No exercise**			
			81 (37.3%)		111 (51.2%) 25 (11.5%)			

Values expressed as mean ± Standard Deviation (95% Confidence Interval) and absolute percentages.

Analysis of the relationship between different outcome variables reported by a clinical examination of the pelvic floor is shown in Table 5.

**Table 5:** **Correlation between outcomes informed by the clinician (n = 208)**

		**Functional capacity: Perineometer assessment**	**Functional capacity: Manual testing right levator ani**	**Functional capacity: Manual testing left levator ani**	**Functional capacity: Strain reflex contraction**
**Functional capacity: Perineometer assessment**	Pearson correlation	1	0,844^**^	0,855^**^	0,021
	*p*		0,000	0,000	0,807
**Functional capacity: Manual testing right levator ani**	Pearson correlation	0,844^**^	1	0,952^**^	−0,066
	*p*	0,000		0,000	0,348
**Functional capacity: Manual Testing left levator ani**	Pearson correlation	0,855^**^	0,952^**^	1	−0,139^*^
	*p*	0,000	0,000		0,047
**Functional capacity: Strain reflex contraction**	Pearson correlation	0,021	−0,066	−0,139^*^	1
	*p*	0,807	0,348	0,047	

**Significative correlation *p* ≤ 0,001.

*Significative correlation *p ≤* 0,05.

The analysis of the relationship between the different outcome variables reported by the patients and those reported by a clinical examination of the pelvic floor is shown in Table 6.

**Table 6:** **Correlations between outcomes reported by the patients and outcomes informed by the clinician**

		**Functional capacity: Perineometer assessment**	**Functional capacity: Manual testing right levator ani**	**Functional capacity: Manual testing left levator ani**	**Functional capacity: Strain reflex contraction**	**SF-12 PCS**	**SF-12 MCS**	**EuroQol (Index)**	**EuroQol (VAS)**
**Functional capacity: Perineometer assessment**	Pearson Correlation	1	-,064	-,066	-,132	,076	-,396^*^	-,142	-,023
	*p*		,719	,675	,400	,654	,015	,371	,886
**Functional capacity: Manual Testing right levator ani**	Pearson Correlation	-,064	1	,821^**^	,838^**^	,118	,112	-,168	,114
	*p*	,719		,000	,000	,541	,562	,350	,528
**Functional capacity: Manual testing left levator ani**	Pearson Correlation	-,066	,821^**^	1	,983^**^	,009	,063	-,172	,194
	*p*	,675	,000		,000	,956	,712	,282	,224
**Functional capacity: Strain reflex contraction**	Pearson Correlation	-,132	,838^**^	,983^**^	1	,010	,110	-,197	,198
	*p*	,400	,000	,000		,955	,516	,217	,214
**SF-12 PCS**	Pearson Correlation	,076	,118	,009	,010	1	-,223	,494^**^	,298
	*p*	,654	,541	,956	,955		,178	,002	,077
**SF-12 MCS**	Pearson Correlation	-,396^*^	,112	,063	,110	-,223	1	,239	,310
	*p*	,015	,562	,712	,516	,178		,161	,066
**EuroQol (Index)**	Pearson Correlation	-,142	-,168	-,172	-,197	,494^**^	,239	1	,275
	*p*	,371	,350	,282	,217	,002	,161		,074
**EuroQol (VAS)**	Pearson Correlation	-,023	,114	,194	,198	,298	,310	,275	1
	*p*	,886	,528	,224	,214	,077	,066	,074	

**Significative correlation *p* ≤ 0,001.

*Significative correlation *p ≤* 0,05.

SF-12 PCS. Physical Component Summary.

SF-12 MCS. Mental Component Summary.

## Discussion

The main contribution of this study is the stratification of the strength of the pelvic floor muscle (PFM) in a normal distribution of a large sample of women with SUI, allowing reference baseline data for the clinic in order to have a preventive paper and to establish levels of severity. We also found significant relationships between the values obtained by evaluating the manual strength assessment by the therapist and the values found by the perineometer. Another important point is the absence of a significant relationship between muscle strength and informed quality of life values of patients.

Most of the studied women had a muscular strength of three or lower on the Oxford Scale in both right and left levator ani muscles, which makes the average strength being in the moderate range. This fact is consistent with a previous epidemiological study developed in 1,732 Spanish women [25], where strength was evaluated by manual palpation. However, our results are not consistent with those presented by Ferreira et al. [22], who studied a group of students with a mean age less than that presented in our study and whose subjects were nulliparous.

Moreover, the average values of muscular strength reported by the perineometer were moderate (seven on a 0–12 scale) and most of the patients were allocated between quartiles 2 and 4. These findings agree with Isherwood and Rane [24] who showed a similar value of strength in a group of 210 patients with previous deliveries; they are in contrast with the values of 59 nulliparous women. According a recent study the pelvic floor muscle function of multiparous women was lower than that of nulliparous women, regarding electrical activity andmuscle strength [26].

The current study shows a high correlation between values obtained with the “perineometer” and the manual evaluation of the PFM, matching with Isherwood and Rane [24] who used the same systems for the assessment of the PFM and reported an agreement between the two systems (kappa value 0.73). Further, Morin et al. [27] obtained significant correlations between the two ways of measurement in groups of continent and incontinent women r =0.727 and r =0.450, respectively (*p* < 0.01). Other previous work suggested a good correlation between the maximum pressure obtained using a perineometer and manual palpation with the Brink Scale [26,28]. However, Bø and Finckenhagen [29] found no significant differences between manual assessment and the value obtained with the pressure manometer, but they defend manual palpation as an important method in pelvic floor rehabilitation.

This study also has consistent values in terms of the quality of life measured by the SF-12 questionnaire with a previous study [30], which evaluated a sample of 312 Spanish women aged between 50 and 64 years who showed very similar means in physical and mental scales. The results also agree with Fialkow et al. [31], who investigated a population of 342 women with urinary incontinence. The quality of life, as assessed by the EQ-ED questionnaire, showed results that matched with a sample of Spanish patients included in a study [32] of 9,487 women with UI in 15 European countries; however, our results are lower where compared with other countries included in the analysis [33]. This fact suggests a poorer self-perceived quality of life in the Spanish women, which could be related to the lack of information and understanding by health professionals and the environment. This could also explain the lack of correlation between functional variables and the values reported on the impact on quality of life.

The mean age of the women studied was 39.93, as opposed to a previous study that established a mean age of 43.39 in the Spanish population of women with UI [32]. However, it is lower than the 55.8 and 54.32 mean ages presented by Espuña-Pons in 2004 and 2007 [24,34], respectively. The BMI of our sample, 24.49, is in accordance with a previous study in Spanish women [33], but is lower than in other studies that show BMIs of 25.53 [35] or 27.54 [24].

The average of 1.56 deliveries agrees with previous studies showing 1.29 deliveries in the Spanish population [33], but is slightly lower than the 2.2 shown in other studies [29,36]. Eighteen per cent of the patients had an instrumented delivery, which differs slightly from a previous study showing that 5.8% of deliveries were instrumented in a population of 243 women from Taiwan [37]. The rates of episiotomy also contrast with a previous study [36], which showed that 100% of deliveries used episiotomies, as opposed to 26.6% in our study. There is evidence that women who had a vaginal delivery were more likely to suffer from urine loss during and after pregnancy than those who had a caesarean [38,39].

Almost half of the sample suffered urine loss while exerting effort, and it was in situations such as coughing, laughing, jumping, hearing the sound of water or after coitus. These data are partially consistent with those reported in a previous study [40] developed in a sample of 154 women with UI: 26% reported urine loss while exerting great effort, 86.4% with coughing, laughing or sneezing, 28.6% during fast walking or running. The average number of micturitions during the day was 7.4, and 0.8 was the average number of nocturnal micturitions. These numbers do not fully agree with the study of Martinez-Córcoles et al. [40], who reported that 60% of patients urinate every 60–120 minutes at night: about three times on average. Almost half of the patients concerned had to wear pads to cope with the loss of urine, and the mean number of pads used for were 2.38 daily. These results are not consistent with a previous study [41] in which 82.5% of patients used fewer than six pads daily, although we have no data if they were used as need or for prevention.

Among the limitations of this study is the fact that we do not have all the socio-demographic data of the study population. Other aspects, as pelvic floor dysfunction could be included in the interview and SUI specific questionnaires in future studies. This is due to the sample selection, which was made directly through the Consulting Unit of Physiotherapy of the Pelvic Floor, and which had to follow the dynamics posed for inclusion of patients in this service. However, the authors consider that the volume of descriptive data presented is very important since many aspects of previously validated questionnaires were collected and can be referenced by future studies looking to make more extensive comparisons regarding population subgroups. It would be necessary to recruit a stratified sample to export the results to the general population.

This paper adds important findings in terms of establishing cut-off points of patient groups according to the strength of the PFM and the relationship of clinical variables obtained by the physiotherapist and those reported by the patients. Future studies should be carried out in order to analyse the strength of the PFM before and after an intervention and to determine the sensitivity to change of the strength of the pelvic floor muscles.

## Conclusion

The strength of PFM has been stratified in a normal distribution of a large sample of women with SUI creating a baseline for the clinic, to both prevent and establish levels of severity. There is a relationship between the strength of the pelvic muscles assessed manually and that obtained by a perineometer in women with SUI. There is no relationship between these values of strength and general health and quality of life perceived by them.

## Additional file

Additional file 1:
**Pelvic floor assessment.**
