# Supplementary material for: Normal reference values of strength in pelvic floor muscle of women: a descriptive and inferential study
Source: BMC Womens Health. 2014 Nov 25;14:143. doi: 10.1186/s12905-014-0143-4 (PMC4251926; doi:10.1186/s12905-014-0143-4)
Supplement: Additional file 1: — Pelvic floor assessment. [file 12905_2014_143_MOESM1_ESM.docx]

**PELVIC FLOOR ASSESSMENT**

Date:

Name, Surname:

Age:

Address:

Telephone number:

1. Group: A B C

1. Prolapse:

Bladder Yes No

Womb Yes No

Rectum Yes No

1. Perineometer assessment:
2. Manual Muscle Testing of levator ani:

Right: 1 2 3 4 5

Left: 1 2 3 4 5

1. Strain reflex contraction: Yes No
2. Numbers of pregnancies and deliveries: 1 2 3 4 5 More
3. Date of delivery: Labour 1: Labour 2: Labour 3: Labour 4: Labour 5:
4. Type of delivery: Natural Instrumented Caesarean

Labour 1:

Labour 2:

Labour 3:

Labour 4:

Labour 5:

1. Weight at birth:

Labour 1:

Labour 2:

Labour 3:

Labour 4:

Labour 5:

1. Multiple birth:

Labour 1: Yes No

Labour 2: Yes No

Labour 3: Yes No

Labour 4: Yes No

Labour 5: Yes No

1. Episiotomy: Yes No
2. Number of micturition per day: < 5 5 to 8 > 8
3. Number of micturition per night: 1 2 or 3 > 3
4. How much fluid do you ingest per day?

< 1 litre 1 litre >1 litre

1. What type of drinks do you have?
2. Have you reduced your ingestion of fluids per day? Yes No
3. Do you suffer from loss of urine during the day or night? Yes No
4. Does it happen during strain or stress? Yes No
5. Do you suffer from constipation? Yes No
6. Do you lose your urine:

a. with coughing, laughing or jumping

b. listening to the sound of water

c. after coitus

1. Do you often feel a strong and urgent desire to urinate? Yes No
2. Do you need to use pads to solve your problem?

If yes, how many?

1. Do you suffer from a loss of sediment?
2. Occupation/Employment:

a. Standing

b. Sitting

c. Carrying weights

1. Does this problem affect your quality of life?
2. Have you discussed it with your relatives and friends?
3. Have you discussed it with a specialist?

1. When you feel the desire to urinate, is it always difficult to control? Is it urgent or an emergency?
2. Has any member of your family had prolapses, bladder surgery, a hysterectomy or urinary incontinence?
3. Do you suffer from anal incontinence?

a. I cannot hold gas

b. I cannot hold sediment

1. During sexual intercourse, do you suffer or complain of:

a. Pain

b. Loss of urine

1. Have you experienced menopause: Yes No
2. Age menopause began:
3. What diseases do you suffer from?
4. Have you had any surgeries?
5. Do you have low abdomen heaviness at the end of the day?
6. Do you have a diagnosis of prolapse:

a. Bladder

b. Womb

c. Rectum

1. Do you practice any physical activity? What kinds?
